# Epicureanism and euthanasia

**DOI:** 10.1007/s11017-024-09684-0

**Published:** 2024-09-22

**Authors:** Jeremy W. Skrzypek

**Affiliations:** Ohio Dominican University, 1216 Sunbury Rd., Columbus, OH 43219 USA

**Keywords:** Euthanasia, Physician-assisted suicide, Death, Epicurus

## Abstract

If Epicurean arguments for the harmlessness of death are successful, then they also successfully undermine a common justification for physician-assisted suicide, euthanasia, and the termination of hopeless pregnancies that I call the ‘Mercy Intuition', according to which, by ending the life of a suffering loved one for whom there is little to no chance of recovery, one is relieving that person of her suffering, and thus providing a great benefit to her. For, if death is not a harm to the person who dies, then it cannot be a benefit to her either, even in cases of intense and prolonged suffering. Along these lines, in this paper, I defend the claim that death cannot provide a benefit to those who are suffering. I begin by highlighting the Epicurean foundations of the argument, focusing on three main Epicurean arguments for the harmlessness of death and their no-benefit analogues. I then move on to explore several important limitations of the argument, which make available a number of strategies for avoiding its conclusion. Along the way, I respond to each of these avoidance strategies. I conclude that even granting several of its limitations, the argument still poses a serious challenge to the Mercy Intuition.

## Introduction

It is a commonly held belief that ending the life of a suffering loved one for whom there is little to no chance of recovery is an act of beneficence, an act of mercy. Indeed, to prolong that loved one’s suffering by keeping her alive in such a state would strike many as self-serving, fruitless, and cruel. In ending the life of a suffering loved one for whom there is little to no chance of recovery, one is relieving that person of her suffering, and that is a great good and benefit to her. One reasons that it would be much better for one’s loved one if her life were to end early rather than after enduring additional and unnecessary suffering.

I call the commonly held belief just described the ‘Mercy Intuition.’ Something like the Mercy Intuition often serves as an important element in the justification of euthanasia, physician-assisted suicide, and the termination of what one might call ‘hopeless pregnancies’—pregnancies in which, due to severe fetal abnormalities, the child’s life is expected to be very short (if it survives to term at all) and extremely painful.^1^ It also often underlies the difficult decision(s) that many make to end the lives of their suffering pets. In this essay, I will defend the claim that the Mercy Intuition, while noble in its intention, is misguided. Indeed, I will defend the position that the Mercy Intuition is not only false, but also, given certain other assumptions, incoherent.

The argument for this conclusion is simple and straightforward. Barring the existence of an afterlife, ending a suffering subject’s life means terminating that subject; one is bringing it about that the suffering subject no longer exists. Now, plausibly, something is only a benefit to some subject if that subject is, at least in principle, capable of receiving it. At the very least, there must be an existing subject to be benefitted. Similarly, one state of affairs can only be better for some subject than some alternative state of affairs if the subject exists in both states of affairs. A state of affairs that does not include the subject can be neither good nor bad, better nor worse for that subject. And so, if ending the suffering subject’s life means terminating that subject, making it the case that that subject no longer exists, then it cannot be a benefit to that subject. Similarly, if the state of affairs in which one terminates the subject’s life is one in which the subject no longer exists, then that state of affairs cannot be better for that subject than any state of affairs in which she continues to exist, even those involving intense and prolonged suffering.

Readers familiar with contemporary debates on the philosophy of death will notice the structure of this argument. It is an Epicurean argument, based on Epicurean arguments for the harmlessness of death, only reversed.^2^ Instead of emphasizing the purported harm of death, it emphasizes the purported benefit of death for those who suffer. I am not the first to make the connection between Epicurean arguments for the harmlessness of death and rational justifications for suicide, physician-assisted suicide, euthanasia, the termination of hopeless pregnancies, or the killing of one’s suffering pets.^3^ Epicurus himself may have even noticed some of these potential applications of his argument.^4^ Nonetheless, I think the argument is important and worthy of a wider audience. And so my aim here is to extend it and defend it.

I begin in the next section by highlighting the Epicurean foundations of the argument. Here I summarize three different Epicurean arguments for the harmlessness of death and introduce a no-benefit analogue for each. I then move on to explore several important limitations of the main argument of this essay, limitations which make available several strategies for avoiding its conclusion. Along the way, I respond to each of these avoidance strategies. I conclude that even granting several of its limitations, the argument still poses a serious challenge to the Mercy Intuition, an important element in common justifications for physician-assisted suicide, euthanasia, the termination of hopeless pregnancies, and the killing of one’s suffering pets.

## Three Epicurean arguments

In his oft-quoted *Letter to Menoeceus*, Epicurus famously argues that death is not and cannot be any kind of harm to the person who dies. He argues as follows: “death, the most frightening of bad things, is nothing to us; since when we exist, death is not yet present, and when death is present, then we do not exist. Therefore, it is relevant neither to the living nor to the dead, since it does not affect the former, and the latter do not exist” [20, p. 29]. Lucretius, a later Epicurean, offers another famous argument for the same conclusion in his *On the Nature of Things*: “Look back now and consider how the bygone ages of eternity that elapsed before our birth were nothing to us. Here, then, is a mirror in which nature shows us the time to come after our death. Do you see anything fearful in it?” [21, p. 94]. There are at least three different arguments for the harmlessness of death that can be derived from these Epicurean texts.^5^

The first is the no-subject argument. The no-subject argument asks one to consider for whom a person’s death might be a harm. Naturally, one wants to say that a person’s death is a harm, if to no one else, then at least to the person who dies. But if, at death, a person ceases to exist (following [24], I will call the claim that at death a person ceases to exist, the ‘termination thesis’), then it seems that death cannot be a harm to that person, and this is so because the person no longer exists to be harmed.^6^ If, for every harm, there must be someone or something who is harmed, then it is also plausible to suggest that death is not and cannot be a harm.

Similar to the first, the second Epicurean argument for the harmlessness of death is the no-time argument.^7^ The no-time argument asks one to consider at which moment or during which temporal interval a person’s death might be a harm. It seems that there are at least three possible answers: a person’s death might be a harm to her sometime before it occurs,^8^ at the exact moment that it occurs,^9^ or sometime after it occurs.^10^ But it seems that death cannot be a harm before it occurs, for death is not yet there to harm the person. And it seems that death cannot be a harm after it occurs, for the person is no longer there to be harmed. And to speak of the exact moment that death occurs is ambiguous: does one mean the very last moment of the person’s being alive or the very first moment of the person’s being dead? For there is no moment at which the person is both alive and dead. And so the answer that a person’s death is a harm to her at the exact moment of death collapses into one of the other two answers. As a result, death cannot be a harm to the person who dies any time before it occurs, nor at the exact moment it occurs, nor any time after it occurs. Therefore, there is no time at which death is a harm for the person who dies. Now presumably, if something harms a person, then there must be some *time* at which it harms her.^11^ And so if there is no time at which death is a harm for the person who dies, then death is not a harm for the person who dies.

The third Epicurean argument for the harmlessness of death is the symmetry argument. Assume that at death I cease to exist. Why do I regard my death as bad, as something that could harm me? Presumably, because it will mean my non-existence, and non-existence is very bad, a serious harm. But consider this. I did not always exist. Indeed, prior to the year of my birth, I neither enjoyed nor suffered any existence whatsoever. But was that era of my non-existence bad for me? Was I, or am I now, harmed at all by my non-existence during this period? Do I look back at those years and regard them as somehow unfortunate precisely because they were years during which I did not exist? It seems not. But if my non-existence prior to the year of my birth was not bad, was no harm to me, then why should my non-existence at death be something very bad, a serious harm to me? Why should it be regarded as something bad at all?^12^

The literature discussing these arguments is vast and complex. Numerous responses have been offered to each of them.^13^ But an underappreciated aspect of these arguments is that their conclusions work both ways: they can be used to argue that one can neither be harmed *nor benefitted* by death.^14^ For if there is no subject to be harmed when death is present, then there is no subject to be benefitted either. And if there is no time as which death harms the person who dies, then there is no time at which it can benefit her. And if my future non-existence is no more a harm to me than my past non-existence, then it is no more a benefit to me than my past non-existence either. The lesson here is that Epicurean arguments for the harmlessness of death, if successful, undermine both negative and positive evaluations of death for the person who dies.

If successful, then, Epicurean arguments for harmlessness of death also successfully undermine the Mercy Intuition. For if the suffering subject ceases to exist at death, then she can be neither harmed nor benefitted by that death. While it is true that her suffering ceases, so does she. She no longer exists to enjoy or be benefitted by the cessation of her suffering. Here one can also extend the argument to include the comparative notions of better for and better than. If the suffering subject cannot be benefitted by the cessation of her suffering due to the fact that upon the cessation of that suffering she no longer exists, then the state of affairs in which her life is cut short cannot be better for her than the state of affairs in which she continues to suffer. And this is so because she does not exist in that first state of affairs. And a state of affairs in which a subject does not exist cannot be good or bad for that subject, nor can it be better or worse for her than any state of affairs in which she does. So not only do Epicurean considerations support the conclusion that death cannot harm the person who dies, they also support the conclusion that death cannot benefit her either.

## Clarifications, limitations, and a variety of ways out

The argument just presented is designed to show that death cannot be a benefit to the person who dies and so it cannot be better for any suffering subject to have its life cut short, which in turn shows that the Mercy Intuition is mistaken. The argument has several notable limitations, limitations which leave open various strategies to those who would try to capture the spirit, if not the letter, of the Mercy Intuition. In this section of the essay, I articulate and respond to several of these limitations and their corresponding avoidance strategies. But first, a clarification.

The Epicurean-inspired argument outlined above asserts that death can neither provide nor threaten neither harm nor benefit to the person who dies. Importantly, the argument neither asserts nor entails that suffering is somehow good or beneficial to the person who carries on in her suffering state. Nor does it assert or entail that suffering somehow fails to harm the person who suffers. The argument above is perfectly consistent with the claim that suffering is bad and harms the person who suffers. What the argument denies is the further claim that the cessation of that suffering, the removal of that harm, provides a good or benefit for that person when it also ends the life of the person. The reason that the cessation of the person’s suffering cannot provide a good or benefit for her when it also ends her life is, once again, because the removal of that suffering also removes the person who suffers. And so the result of the removal of the person’s suffering is that there is no person remaining who could be said to enjoy or experience or in any way benefit from the removal of that suffering. The argument is perfectly consistent with the claim that in ordinary circumstances, removing a person’s suffering provides a great good for that person, that it benefits that person immensely. It only asserts that in the very specific scenario in which doing so also removes that person, it fails to provide the great good, the benefit, that it ordinarily provides. That’s the Epicurean position: suffering is a harm for the person who suffers, but removing that person’s suffering by removing the person provides no benefit to her.^15^

Having made this important clarification, I will now move on to discuss several limitations of the argument. First, the argument above assumes that there is no afterlife, and so the conclusion can be avoided if one rejects that assumption. If one is certain that one’s loved ones do not cease to exist at death but continue to live on, free from harm, in a pleasant or neutral or simply less painful afterlife, then the cessation of their suffering could be a benefit to them.^16^ For, in that case, those loved ones are in a position to experience and enjoy or to otherwise benefit from the freedom from suffering that one’s actions have brought about for them. This way of avoiding the conclusion is not without cost, however. If one were certain that all of one’s loved ones did not cease to exist at death but continued to live on, free from harm, in a pleasant or neutral or simply less painful afterlife, then one ought to wonder if it would not have been better to end their lives sooner. Indeed, if one were certain of the existence of such an afterlife for one’s loved ones, and if one were primarily concerned about their good rather than one’s own, it would seem to follow that one should end their lives very early on, at the first sign of harm, to prevent them from ever having to suffer at all.

But what if one rejects the claim that at death a person ceases to exist, not because one thinks that there is some kind of afterlife, but because one accepts a bodily continuity theory of personal identity, according to which the person is identified with her body, living or dead [24]. In that case, under normal circumstances, circumstances in which the person’s body has not been obliterated, consumed, or dissolved, the person who dies is still around for death to be a benefit to her.^17^ Once a living organism, the person is now a corpse. And, as a corpse, the person suffers no more. She is at peace, preserved from any kind of pain or suffering, relieved of any of the stresses and anxieties of life. But I wonder if this solution actually solves the problem. If the termination thesis is false, and the bodily continuity theory is true, and a person continues to exist after her death as a corpse, then the person in such a state would be neither conscious nor aware nor capable of any kind of sensory experience. But if the person is neither conscious nor aware nor capable of any kind of sensory experience, then it is hard to see how her death, and the lack of suffering that it provides, could be something that she enjoys or experiences or otherwise benefits from. Upon her death, the person no longer possesses any kind of capacity for sensation, growth, reproduction, or conscious experience. Indeed, upon her death, it would seem that the person no longer has any interests or desires whatsoever. And so it is hard to see how death (or anything else) could be any kind of benefit or harm to her in such a state. A corpse just does not seem like the sort of thing that can be meaningfully benefited or harmed.^18^

A third limitation of the argument is that the conclusion only applies to the *death* of the person, not to the *dying* of the person. If what I have argued is correct, then death cannot be a benefit to the person who dies, for the simple reason that when death arrives, the person is no longer there to be benefitted by it. Death cannot be a benefit to the person who dies because it is not something that the person ever sees or experiences. Dying, however, is something that a person often sees or experiences. Dying is the process by which a person approaches death, it is the slowing or receding or ceasing of the life of that person. According to the termination thesis, a person ceases to exist at death. Presumably, then, a person remains in existence up until the very moment of death. And so, she is there during the entire process of dying. Now, because the person is present while she is dying, it is possible for that process to be a harm or a benefit for her, depending on the nature of that dying. It could be a time of reconciliation and peace or it could be a period of anguish and dread. It could be a period of great suffering or it could be a period of great joy. Whether it is one or the other will depend on her theory of value and the circumstances of her dying. But the important point is that since the subject is there to experience her own dying, it can in principle be a harm or a benefit to her. The Epicurean argument above does not in any way block or support either conclusion.^19^

A fourth limitation of the argument, which leaves open another possible way around its conclusion, is that it only applies to the suffering subject herself. I have argued that, if, at death, the suffering subject exists no more, then the cessation of her suffering cannot be a benefit to her, for she no longer exists to be benefitted. And, if so, it cannot be better for her to have her life ended early, because the state of affairs in which that takes place is one that no longer includes her. However, the cessation of the subject’s suffering can be a benefit to others, and the state of affairs in which her life is ended early can be a better state of affairs for those who survive her. Her loved ones no longer have to experience the pain and grief of watching their loved one suffer or carry the burden of caring for their loved one while she suffers, and this is a benefit that they can enjoy by ending her life early. Those whose lives could be saved by the resources made available by her early death can also enjoy the benefits of those resources. But to say that the suffering subject herself is benefitted or made better by the cessation of her suffering is a mistake. Despite what her loved ones might think, and despite how they might rationalize their decision to end her suffering by ending her life, they are not acting for her sake. They are not doing it to provide some benefit to her. The only subjects who are still around to be benefitted by her death are her loved ones and those whose lives can be saved by the resources made available by her early demise. And so, if her loved ones are acting for the benefit of anyone, it seems that they can only be acting for their own benefit, or the benefit of those whose lives can be saved by the resources made available by her death. And that is a far cry from the act of mercy or act of beneficence that they intended to perform by ending her life.

But could not one say that the cessation of one’s loved one’s suffering is a great good not for her (and perhaps, in some cases, not for anyone else either) but a great good nonetheless? Is it not good that her suffering has ended? If it is bad that suffering exists, is it not a good thing if there is less of it? Similarly, could not one say that the state of affairs in which the suffering subject’s life is ended early is better than the state of affairs in which she continues to suffer not for her or for anyone in particular, but just better overall? If something can indeed be good without being good for anyone in particular, just plain good, and if some state of affairs can indeed be better than some other state of affairs without being better for anyone in particular, just plain better (say, because it includes less suffering, or fewer instances of suffering, or fewer suffering people), then, yes, the cessation of the suffering subject’s suffering can be good, and the state of affairs in which the suffering subject’s life is ended early can be considered a better state of affairs than the state of affairs in which she continues to suffer.^20^ But notice that this is a very different kind of argument. To shift to this kind of argument to justify ending the suffering subject’s life is already to give up on the idea that one can justify the ending of the suffering subject’s life by reference to some good or benefit for her. And that the ending of the suffering subject’s life provides some good or benefit for her is precisely what allows one to classify that act as one of mercy or beneficence in the first place. It is what underlies the sentiment that one is doing this not for one’s own good, or for anyone else’s, but for hers.

Here's one final way around the conclusion of the argument. Compare the suffering subject’s entire life, a life that includes the extended period of intense and prolonged suffering, to a version of that subject’s life that is exactly the same except that it is cut short and so excludes the extended period of intense and prolonged suffering. Is the latter version of her life not better than the former, since it includes less suffering? Is it not a better life for her? Would not allowing her to live the better life rather than making her live the worse life be a good thing, a benefit, to her? This response is a variation of the standard deprivationist response to Epicurean arguments for the harmless of death.^21^ According to deprivationist accounts of the harm of death, when death harms a person it harms her precisely because it deprives her of the valuable goods that she would have experienced had she not died, or alternatively, because it deprives her of the opportunity to live the version of her life that is, overall, better. And when death benefits a person it benefits her precisely because it preserves her from certain evils that she would have experienced had she kept on living, or alternatively, because it preserves her from living the version of her life that is, overall, worse.^22^

However, I think that the deprivationist response fails, and for reasons similar to those articulated above. Even if it were true that the longer life with more suffering is in some way worse than the shorter life with less suffering, it cannot be a worse life for the subject. Allow me to demonstrate. Compare two possible “lives” that a person could live:
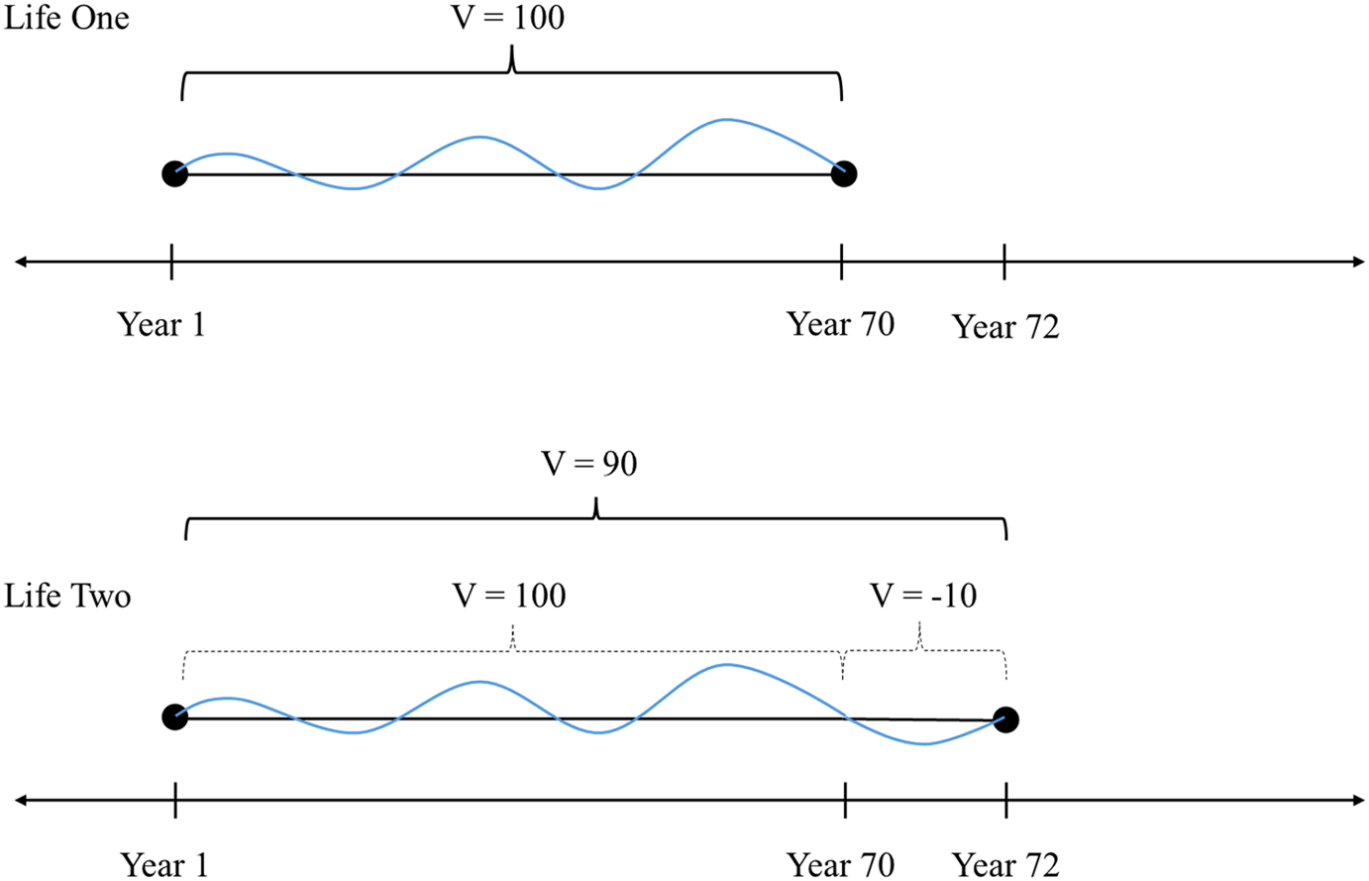


The first version of this person’s life has a duration of seventy years and includes the normal ups and downs of life. The ups are higher and more frequent than the downs and so, overall, the life has a net positive value. The second version of the person’s life has a duration of seventy-two years. The first seventy years of the second life are identical to the first life. The only difference between the two is what takes place during the additional two years not contained in the first. During those final two years, the second life includes periods of intense suffering which decrease the overall value of the person’s life, but not so much as to produce a net negative value for that life overall. Now, assume that the overall value of the first life is 100 points and that the periods of intense suffering during the last two years of the second life decrease the overall value of that life to 90 points. But now break the value of those lives down further. Since both lives have the same value for the first seventy years but only differ in the last two, the only period during which one life could be better than the other is after that point. But, crucially, on the timeline for the first life, the timeline in which her life is supposed to be better for her, the person no longer exists during the only times at which it is better. And so even if her ‘life’ is better in some way from this point on, it seems that it cannot be better for her, since she no longer exists.^23^

Now, if the person’s life is not better for her prior to the point of divergence (since the two lives are exactly the same), and it is not better for her after the point of divergence (given that she no longer exists at all), then at no point is that life better *for her*. As a result, one is left with the puzzling conclusion that one of the versions of her life is indeed better than the other, though there is no time at which it is better for her. Ending her life early as a means of ending her suffering is no benefit to the suffering subject since there is, once again, no time at which she benefits from it. It seems that the only way that one of those lives could be better for her would be if the extended period of intense and prolonged suffering were to somehow backtrack and decrease not just the overall value of the life but the value of the earlier period. For then one could say that one of the lives is better for her than the other, because one would be speaking of the period during which she exists in both. But notice how strange that would be. Can later events in one’s life somehow decrease the value of events which have already taken place? Is that not a kind of backwards causation? How can value adjustments travel back in time that way?^24^

## Conclusion

In summary, Epicurean arguments for the harmlessness of death, if successful, undermine both negative and positive evaluations of death for the person who dies. Death can neither harm nor benefit the person who dies. But if death can neither harm nor benefit the person who dies, then ending the life of a suffering loved one for whom there is little to no chance of recovery provides no benefit to her. It does not bring about a better state of affairs for that person than the alternative of allowing her to suffer longer. This is a surprising and counterintuitive result. It calls into question a commonly held and intuitively plausible belief that I have called the Mercy Intuition, which is a crucial source of consolation upon which many rely during and after difficult end of life decisions concerning terminally suffering loved ones. As I explained above, the argument defended in this paper has several important limitations. It assumes the truth of the termination thesis. It leaves open the possibility that the person’s death could be a great good or benefit to others or a great good but not for anyone in particular (and so leaves standard utilitarian justifications for ending the lives of terminally suffering patients completely intact). And it does nothing to undermine the claim that suffering and dying are harmful to anyone who experiences them. But, for all that, it still provides an important challenge to how many ordinarily think about these issues. Despite what one might want to believe, ending the life of a suffering loved one for whom there is little to no chance of recovery does that loved one no good. Her early death, despite the fact that it puts an end to her suffering, provides no benefit to her. And so, if one is to justify ending that person’s life, one must justify it by other means. Appeals to mercy will not do the job.

## Footnotes

1 See, for example, [1–3].

2 Recent defenders of Epicurean arguments for the harmlessness of death include among others, Rosenbaum [4–7], Hershenov [8], Smuts [9], Taylor [10, 11], Pavelich [12], and Suits [13, 14].

3 See [15], [10, pp. 106–109], [16, p. 465], [17], [12], and [14, pp. 204–206].

4 Epicurus himself seems to have suffered for several years leading up to his death and still never considered ending his own life [18, 19].

5 For helpful overviews of these three arguments, see [22, 23]. Cyr and Fischer also draw out a fourth possible argument—a no-experience argument—from these texts, but I will focus on just the following three.

6 Notice that the claim here is not that a person must know about or feel the effects of something in order for it to count as a harm to her. I do not think that the Epicurean has to be committed to that claim. And it is a good thing too, since that claim seems too strong. It is plausible to suggest that I can be harmed by a bad reputation that I do not know I have or an underlying condition that has yet to be diagnosed. All that this argument claims is that a person must exist in order to be harmed. Understood as a condition or state, harm requires some subject, something that is in that condition or state of harm. A person can be harmed or be the subject of some harm without knowing about or feeling the effects of that harm, but she cannot be harmed or be the subject of some harm if she does not exist as a possible subject of harm. McMahan [25] calls this the “Existence Requirement” and rightly distinguishes it from the “Experience Requirement.”.

7 For helpful overviews that concentrate on this particular argument, see [26, pp. 122–139] and [27].

8 This view is known in the literature as “priorism.” Proponents include Pitcher [28], Feinberg [29], Li [30, 31], Luper [26, 32]. The trick that priorism needs to pull off is to explain how some future event could reach back into the past and cause harm to some person in the present, or, more charitably, how it could be true that whether some present event constitutes a harm to some particular person depends on events that have not yet occurred.

9 This view is known in the literature as “concurrentism.” Proponents include Lamont [33] and Wolf [34].

10 This view is known in the literature as “subsequentism.” Proponents include Feit [35–37], Bradley [38–40], Grey [41], Purves [42], and Fischer [43]. The trick that subsequentism needs to pull off is to explain how one can award a positive or negative (or neutral) level of well-being to a person at a time at which that person does not exist. Johansson [27] also explores two other possibilities: “eternalism,” according to which death harms the person at all times [44, 45, p. 154], and “atemporalism,” according to which death harms the person but not at any time [46–48]. Johansson [27] argues for the atemporalist position. The trick that eternalism needs to pull off is to explain how a person can be harmed not only after she is gone but also before (and long before) the first moments of her existence. The trick that atemporalism needs to pull off is to explain how a person who exists in time can suffer some harm, and yet not suffer that harm at any time during which she exists. If the person is in time and the harm is not, where do the two meet?

11 This is the key premise in the argument that the atemporalist denies.

12 Note here that the primary issue is not the psychological question of why humans tend to fear their future non-existence and not their past non-existence, or the rationalization question of why this asymmetrical fear might be rational for other reasons. The primary issue is the metaphysical question of how it could be the case that one’s future non-existence harms him or her but his or her past non-existence does not. While there have been numerous attempts to answer the first two questions (see [49] for a helpful overview), I would argue that many fail to address the real issue at hand.

13 See, once again, [22, 23].

14 A notable exception is [12]. One of the shortcomings of Pavelich’s otherwise excellent article is that he fails to properly distinguish between the three different Epicurean arguments for the harmlessness of death just outlined and their no-benefit analogies. He also does not consider or address (or if he does address, does not adequately address) several of the limitations of the argument that I explore below.

15 And this is where I think Hershenov’s proposed solution does not quite work. Hershenov [8] argues that while death is not a harm to the person who dies, intentionally bringing about that person’s death is, in most cases, morally wrong because it prevents that person from enjoying various goods that she would have enjoyed had she lived longer. The basic idea here is that one need not say that death is bad in order to say that continuing to live is often good. In the case of the suffering subject, on Hershenov’s account, while an early death is not a benefit to that person, causing her death could nevertheless be justified by the fact that it prevents her from experiencing any future suffering. The basic idea here is that one need not say that an early death is good in order to say that continuing to suffer is very bad. In response, I would say that, once again, while it is true that suffering is bad, and that, in most cases, preventing someone’s future suffering is a great good for that person, any act that prevents that future suffering by also removing the person who suffers cannot be justified by reference to some good or benefit to that person, including the good or benefit of having her present suffering removed or future suffering prevented. For, once again, by the time that the suffering that otherwise would have continued has ceased, the person is no longer there to experience or enjoy or in any way benefit from the cessation of that suffering. And so, while the suffering that the person experiences is bad for her, the cessation of that suffering is neither good for her nor better for her than any continued state of suffering. As a result, ending the person’s life early can in no way be justified by reference to some good or benefit to her, including the good or benefit of the removal of any present suffering or the prevention of any future suffering she can be expected to experience. One could argue that the removal or prevention of intense and prolonged suffering is nonetheless a great good, even if it is not a good or benefit for the person who suffers, but that would be a different argument (I say more about this alternative line of argument below). (Thanks to anonymous referee to pushing me to address this point.).

16 Though, all things considered, their death could still be bad or harmful to them. See [50, 51] for more on this.

17 Similarly, one could hold that while the dead do not exist, they still maintain some level of being, which might enable them to continue to serve as a subject for various predicates [52]. My response to the bodily continuity theory reply applies equally well to this proposal.

18 Luper makes a similar point in his [32], arguing that a person in such a state cannot be harmed because she is entirely unresponsive. Once again, the claim is not that someone needs to know about or feel the effects of something in order for it to count as a harm or benefit to her. She just needs to be the sort of thing that can be harmed or benefitted. And it is not clear that a corpse is the sort of thing that can be harmed or benefitted.

19 See [4] and [53] for similar observations.

20 This is a point well-appreciated by Pavelich [12].

21 Deprivationists include, among others, Nagel [46], Brueckner and Fischer [54], McMahan [25], Feldman [44, 45], Bradley [39], Luper [26], Feit [35–37].

22 Notice that the deprivationist account of the harm of death is a counterfactual account. Death is not a harm because of the actual goods that it removes but because of the possible goods or opportunities that it prevents.

23 For similar complaints about standard deprivationist responses, see [55, p. 410], [42, pp. 103–104], [56, pp. 757–758], and [14, p. 101]. One might also wonder whether it is right to speak of the first life as a better life than the second at all, for, once again, the only part of the timeline during which the second life is worse is that part during which the first life has already ceased. And so, at that part of the timeline, both lives are not there to be compared to one another (Thanks to an anonymous referee for this suggestion). There is, however, an important additive or cumulative sense in which the first life turns out to be better than the second: when one tallies up their overall values, one finds that the overall value of the first is higher than the overall value of the second. What I am arguing here is that this additive or cumulative sense can make no difference to the person herself, since she is not present during that part of the timeline in which the relevant values diverge.

24 Even if it is not a kind of backwards physical causation, the suggestion would be that the value of a particular person’s life or the particular experiences enjoyed by the person at an earlier time is determined by or grounded in facts about what is or is not occurring some time in the future. If not backwards causation, then, it is a kind of backwards determination or dependence.
